# Leptin Regulation of Gonadotrope Gonadotropin-Releasing Hormone Receptors As a Metabolic Checkpoint and Gateway to Reproductive Competence

**DOI:** 10.3389/fendo.2017.00367

**Published:** 2018-01-05

**Authors:** Angela K. Odle, Noor Akhter, Mohsin M. Syed, Melody L. Allensworth-James, Helen Beneš, Andrea I. Melgar Castillo, Melanie C. MacNicol, Angus M. MacNicol, Gwen V. Childs

**Affiliations:** ^1^Department of Neurobiology and Developmental Sciences, University of Arkansas for Medical Sciences, Little Rock, AR, United States

**Keywords:** gonadotropes, Musashi1, miRNAs, infertility, female, gonadotropin-releasing hormone receptor, leptin receptors, post-transcriptional regulation

## Abstract

The adipokine leptin signals the body’s nutritional status to the brain, and particularly, the hypothalamus. However, leptin receptors (LEPRs) can be found all throughout the body and brain, including the pituitary. It is known that leptin is permissive for reproduction, and mice that cannot produce leptin (Lep/Lep) are infertile. Many studies have pinpointed leptin’s regulation of reproduction to the hypothalamus. However, LEPRs exist at all levels of the hypothalamic–pituitary–gonadal axis. We have previously shown that deleting the signaling portion of the LEPR specifically in gonadotropes impairs fertility in female mice. Our recent studies have targeted this regulation to the control of gonadotropin releasing hormone receptor (GnRHR) expression. The hypotheses presented here are twofold: (1) cyclic regulation of pituitary GnRHR levels sets up a target metabolic checkpoint for control of the reproductive axis and (2) multiple checkpoints are required for the metabolic signaling that regulates the reproductive axis. Here, we emphasize and explore the relationship between the hypothalamus and the pituitary with regard to the regulation of GnRHR. The original data we present strengthen these hypotheses and build on our previous studies. We show that we can cause infertility in 70% of female mice by deleting all isoforms of LEPR specifically in gonadotropes. Our findings implicate activin subunit (InhBa) mRNA as a potential leptin target in gonadotropes. We further show gonadotrope-specific upregulation of GnRHR protein (but not mRNA levels) following leptin stimulation. In order to try and understand this post-transcriptional regulation, we tested candidate miRNAs (identified with *in silico* analysis) that may be binding the *Gnrhr* mRNA. We show significant upregulation of one of these miRNAs in our gonadotrope-Lepr-null females. The evidence provided here, combined with our previous work, lay the foundation for metabolically regulated post-transcriptional control of the gonadotrope. We discuss possible mechanisms, including miRNA regulation and the involvement of the RNA binding protein, Musashi. We also demonstrate how this regulation may be vital for the dynamic remodeling of gonadotropes in the cycling female. Finally, we propose that the leptin receptivity of both the hypothalamus and the pituitary are vital for the body’s ability to delay or slow reproduction during periods of low nutrition.

## Introduction

### The Leptin Signal Permits Reproduction

Leptin is a hormone largely produced by adipocytes that regulates appetite and signals levels of adiposity and nutritional status (1–8). When physiological conditions are normal, serum leptin levels correlate well with fat mass and signal optimal nutritional states (9–11). When nutrition is deficient, the resulting reduction in serum leptin becomes a critical metabolic signal for starvation (12–17), stimulating increases in appetite and food-seeking behavior. At the same time, the low leptin signal reduces or prevents the activation of energetically expensive reproductive processes such as pregnancy and lactation (3, 14, 18–33).

Serum leptin levels are a critical link between sufficient nutrition and the function of the hypothalamic–pituitary–gonadal (HPG) axis. The importance of leptin to the HPG axis is emphasized by evidence in humans deficient in leptin receptors (LEPRs) (20) or leptin (34, 35), who are hypogonadal and infertile. Furthermore, low gonadotropin levels and functional hypothalamic amenorrhea occur when leptin is reduced by energy deficits caused by weight loss, excessive exercise, or eating disorders. Women with hypothalamic amenorrhea have low leptin levels and do not express the normal diurnal leptin rhythm (19, 22, 24, 36–40).

Leptin therapy normalizes reproductive hormone levels (2) and restores cycles in women with functional amenorrhea (39, 40). Specifically, leptin increases luteinizing hormone (LH) levels and pulse frequency, ovarian volume, serum estradiol, and numbers of dominant follicles (16, 22, 38–41). Leptin’s therapeutic benefit has also been shown in studies of a leptin-deficient prepubertal child (42) and of adult men (43).

Leptin’s role in reproduction has also been modeled in lower mammals. Fasting that lowers serum leptin also reduces pulses of LH in rodents or non-human primates (10, 44–48). Leptin antiserum administered into the ventricular system of fed rats disrupts cyclicity and LH secretion (49). Conversely, leptin treatment increases serum prolactin and LH pulse frequency and amplitude in fasted rats (50, 51).

*In vitro*, leptin treatment of pituitary cells from fasted rats restores LH stores depleted by food deprivation (52). Similarly leptin injections reverse the loss of reproductive function, decrease LH levels, and prolong estrous cycles in mice that are food-deprived for 48 h (2). Exogenous leptin given to leptin-deficient mice also restores fertility (27, 53, 54). Most recently, studies in non-human primates by Sarmento-Cabral et al. have reported that leptin stimulates growth hormone, prolactin, adrenocorticotropin and follicle-stimulating hormone (FSH) secretion from monolayer pituitary cultures derived from two groups of female monkeys (55).

The observation that a threshold level of fat (and, thus, leptin signaling) is required to permit puberty indicates that the leptin signal is vital for the timing of puberty. In fact, early studies showed that leptin accelerates puberty (1, 53, 56), suggesting that it might be a metabolic trigger, although this was disputed by studies that found no correlation between prepubertal serum leptin levels and the timing of puberty in normal rodents (57–59) or primates (60–63). Furthermore, the rise in leptin during development [i.e., during the second trimester in the human fetus (64) or postnatally in rodents (58, 59, 65, 66)] appears to be too early for it to have direct impact as the trigger for puberty (6, 7, 59), although evidence indicates that leptin does play a permissive role in puberty (67).

### The Role of Distinct Leptin-Target Cells throughout the Reproductive Axis

Leptin receptors can be found in cells throughout the HPG axis, and much research over the past two decades has focused on the relative importance of each set of target cells. The preponderance of evidence points to target cells in the hypothalamus as being most critical for mediating leptin signaling for fertility. However, the identity of the target cells has been a subject for investigation. Pioneering studies by McMinn et al. (8). reported that loss of LEPR in 50–75% of hypothalamic neurons caused obesity and glucose intolerance, but fertility and cold tolerance remained normal. This suggests a division of labor in the neurons responsive to leptin, and that LEPR deficiency must be seen in all neurons for the full set of deficiencies.

This presentation will discuss evidence for different groups of LEPR-target cells and build the case for including the pituitary gonadotrope. In fact, we will propose that leptin sets up an active partnership between leptin-responsive neurons in the hypothalamus and leptin-responsive gonadotropes in the anterior pituitary. In the later sections focused on the hypotheses, we will propose pathways that may be activated by leptin to permit reproduction. First, we will discuss evidence for a role for each of these leptin-target cells as responders to leptin’s permissive actions.

### The Case for the Importance of Neuronal Target Cells to Reproduction

Cre-*loxP* deletion of both alleles of the LEPR gene specifically in all neurons resulted in deletion mutant mice that were infertile (8). This important finding supported the original hypothesis that states that the major target cells for leptin’s permissive effects on reproduction were neurons. Because GnRH neurons do not have LEPRs, a number of studies were then initiated to identify leptin-responsive neuronal pathways that regulate GnRH (4, 23, 27, 30, 57, 68–71) and report evidence for leptin interactions with these neurons (2, 4, 14, 31, 72–85). The relative importance of these neuronal pathways was then strengthened by evidence from two laboratories showing that restoration of LEPR in the neurons of LEPR-null mice partially or completely restored fertility (50, 82, 85). Collectively, this led to the view that other leptin-target cells, such as gonadotropes were considered secondary or redundant responders to leptin’s metabolic signals (50, 82, 85).

### The Case for the Importance of Pituitary Gonadotrope LEPR-Target Cells

Gonadotropes reside within the anterior pituitary, synthesize, store, and secrete LH and FSH in a strict temporal order during the estrous cycle, and are stimulated by GnRH. Evidence supporting gonadotropes as leptin-target cells initially came from studies showing that they express functional LEPR (33, 86–93), and that leptin- or LEPR-deficient mice have reduced numbers of gonadotropes (6, 7, 91, 94). Cytophysiological studies showed that leptin modulates the expression and/or secretion of gonadotropins (27, 30, 33, 95–100). Fasting concomitantly reduced levels of serum leptin and numbers of gonadotropes defined by LH stores or GnRH-binding sites (52). Stores of LH were recovered following a 1-h treatment *in vitro* with leptin, which provides supporting evidence for direct interactions of leptin with pituitary gonadotropes (52). Further evidence stems from our report that pituitary LEPR expression varies with the stage of the estrous cycle with the highest expression before the LH surge (33).

In spite of the evidence for leptin interaction with gonadotropes, questions still remained about their importance as metabolic sensors of leptin signals. A recent study tested the role of LEPR in gonadotrope functions in a recent study that used Cre-*LoxP* technology with a genetically engineered line of mice ubiquitously deficient in LEPR (101). In this study, the recombination event restored LEPR selectively in pituitary gonadotropin releasing hormone receptor (GnRHR) target cells and FSH levels were elevated, although fertility was not restored (101). However, lack of fertility may have been secondary to the fact that the hypothalamic neuronal target cells remained LEPR-null and the mice remained morbidly obese. The GnRH pulse signal, which is vital to the pituitary gonadotrope was still lacking (101).

Thus, restoration of leptin signaling to gonadotropes will not rescue leptin’s permissive effects on fertility in a LEPR-null mouse. However, evidence does indicate that gonadotrope LEPR plays a significant role in optimizing fertility. Our studies ablated the signaling domain of LEPR (encoded by exon 17) in gonadotropes *via* Cre-*LoxP* technology and reported a significant impairment of fertility in females (33). Specifically, there was a reduction in the levels of pituitary GnRHR proteins and activin mRNA (in females). Local activin and its downstream pathways are believed to be vital for the synthesis of FSH (102–104). Analysis of fertility showed significant delays in the time to first litter, abnormal estrous cycles, and lower numbers of pups/litter in breeding cages with deletion mutant dams. Gonadotrope LEPR deletion mutant males showed lower GnRHR proteins, but their fertility was unaffected. Thus, loss of the signaling domain of LEPR in gonadotropes appears to cause subfertility selectively in females.

### Ablation of All Isoforms of LEPR in Gonadotropes May Result in Complete Infertility

To strengthen the case for gonadotropes as important LEPR-target cells, we recently produced a more severe ablation of LEPR selectively in LH gonadotropes with methods described in previous studies (33). All animals were handled and cared for under an animal use protocol that was reviewed and approved annually by the UAMS Animal Use and Care Committee.

We used a different floxed line of mice in which *Lepr exon 1* is flanked by LoxP, and Cre-recombinase is driven by the bovine *Lh-beta* promoter. The breeding strategy to produce this line is described in more detail in previous studies in which these Cre-bearing mice were also used (33). The resulting Cre-recombinase ablation removes the region encoding the signal peptide and prevents the translation of all isoforms of LEPR (105). We reasoned that ablation of the signal peptide would have a deleterious effect on the LEPR-receptor population as seen in our previous studies of mice in which *Lepr exon 1* was ablated in somatotropes (106).

The method is as follows. We produced deletion mutants in three breeding cages with F2-generation *Lh-cre* positive females bearing one allele of floxed *Lepr exon 1* (heterozygotes) and Cre-negative males bearing two alleles of floxed *Lepr exon 1*. Females always passed down the Cre-recombinase because the *Lh-cre* is known to be expressed in the testes (33). All mice were of the same FVB strain and at least 3 months of age when they entered the breeding cages. The reproductive competence of the homozygous and heterozygous mutant females was compared with that of females in cages containing control mice of the same strain background (FVB.129P), which had delivered during the same time. As in our previous studies (33), we tested the period that normally produced 3–4 litters in the wild type FVB.129P strain (65–85 days). The time was extended, however, for cages with mutants that produced few (or no) pups.

The three breeding cages of F2-generation heterozygous females produced an average of eight pups/litter, with a normal time-span between litters of 21–22 days. Thus, their productivity was not different from that in the FVB.129P wild type females. This group of females produced the test population of 11 F3-generation mutant homozygous females (bearing *Lh-cre* and two alleles of floxed *Lepr exon 1*) and 2 F3-generation mutant heterozygous females. This test population came from five different F2-generation litters.

Data on the breeding study are summarized in Table 1. Three of the five F3-generation homozygous deletion mutant females showed total infertility, failing to produce pups after 240–281 days of breeding with a proven Cre-negative male. Two homozygous mutant females were fertile although they produced litters slowly (every 30–45 days) compared with FVB.129P females, which produce at 21–22 day intervals. One of these females produced 6 litters and 42 pups in 199 days, with an average litter size that was nearly normal. The other mutant female produced only 4 litters and 19 pups in 199 days, and one of the litters did not survive. Table 1 also shows that the two test F3-generation heterozygous females also showed no evidence of pregnancy with a proven Cre-negative male. Therefore, breeding with these females was stopped after 65 days.

**Table 1:** Deleting all isoforms of leptin receptor (LEPR) in gonadotropes causes infertility.

Female^a^, date of birth	Genotype *Lh-cre*	# Days with male	# Litters	Average # pups/litter	Total # pups
F0, 8/19/2015	*Lepr^exon 1 f/wt^* Heterozygous	65	0	0	0
F6, 8/19/2015	*Lepr^exon 1 f/wt^* Heterozygous	65	0	0	0
F1, 5/21/2015	*Lepr^exon 1 f/f^* Homozygous	199	6	7	42
F0, 6/11/2015	*Lepr^exon 1 f/f^* Homozygous	199	4	5	19
F6, 3/21/2015	*Lepr^exon 1 f/f^* Homozygous	240	0	0	0
F1, 3/21/2015	*Lepr^exon 1 f/f^* Homozygous	240	0	0	0
F0, 1/20/2015	*Lepr^exon 1 f/f^* Homozygous	281	0	0	0

*^a^F3-generation females bearing Lh-cre and one or two floxed alleles of Lepr^exon 1^ were bred with males bearing only floxed alleles of Lepr^exon 1^. These seven females came from five different F2-generation litters and three different breeding cages. 71% of these females showed infertility*.

**Figure 1:**
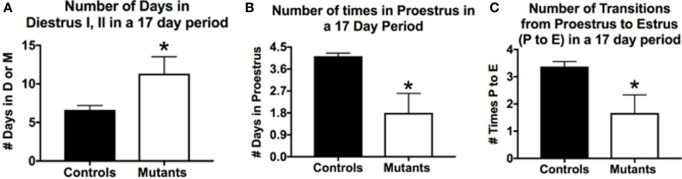
Deletion of *Lepr exon 1* in gonadotropes leads to severe reproductive deficiencies. Deletion mutant mice bearing two floxed alleles of *Lepr* (*Lepr-exon 1^loxP/loxP^* obtained from Dr. Jeffery Friedman) were bred to female mice bearing the *Cre-recombinase* gene driven by the *bLh-*β promoter (*bLh*β-*cre*) developed by Dr. Sally Camper (108). The *Cre-recombinase* was passed through the female line, because it was reported to be expressed in the testes (108), and all breeding was tested only in females. The resulting offspring carried two floxed *Lepr* alleles and were either positive (one allele) or negative for *Cre-recombinase* in gonadotropes. Breeding studies done as described previously (33) produced seven female homozygous mutants that were evaluated for cyclicity over a 17-day period. **(A)** On average, females spent more days in diestrus I (metestrus) or II in the 17-day period days (compared with control females). **(B)** Mutants spent fewer days in proestrus and **(C)** In the 17-day test period, there were only 1.6 proestrous to estrus transitions in the mutants. Stars over mutant bars indicate significantly different values as compared to controls by the Student’s *t*-test.

The breeding generated six F3-generation homozygous mutant females, which could be used in parallel studies of cyclicity, comparing their cycles with those of eight littermate controls bearing no *Cre-recombinase* (Figure 1). We analyzed vaginal smears from these animals daily over a 17-day period with methods described in previous studies (33, 107). Two of the mutant females remained in diestrus during the entire 17-day test period; the remaining 4 showed some degree of cyclicity. The average number of days in diestrus I or II for all mutants was significantly higher compared with controls (*p* = 0.03; Student’s *t*-test; Figure 1A).

In a 17-day test period, one would expect to see 4–5 proestrous days (assuming a 4- to 5-day cycle). Mutants exhibited on average <2 days in proestrus in this test period, which was significantly lower than control values of 4.1/17 days (*p* < 0.03, Student’s *t*-test; Figure 1B). We also evaluated the number of times mice exhibited a proestrus to estrus transition (P–E), which would indicate the completion of a cycle and readiness for copulation during early estrous. Figure 1C shows that controls had 3.4 P–E transitions in a 17-day test period; however, mutants had less than half of these P–E transitions (1.6), which was significantly lower than controls (*p* = 0.01, Student’s *t*-test). Thus, whereas four of the six mutant females cycled, the opportunities for a pregnancy in the 17-day period (seen by the P–E transition) were significantly reduced, which correlates with the low number of litters in the breeding cages of the two subfertile females reported in Table 1.

In conclusion, these data showed a more severe infertility phenotype in mice lacking all isoforms of LEPR in gonadotropes. This resulted in unreliable breeding or infertility, which supports our assertions that gonadotrope LEPR is important to the HPG axis. Analysis of serum levels of gonadotropins and other pituitary and ovarian hormones in ongoing studies will identify the full mechanism behind the loss of fertility in these gonadotrope-*Lepr exon 1* deletion mutants. In spite of the hypothalamic *Lepr* gene remaining intact, gonadotropes having all LEPR isoforms deleted were unable to function normally in most (71%) of the F3-generation female mice tested and preformed sub-optimally in the remaining two mice. The infertile group also included a subset of F3-generation heterozygous mice (which lacked only one allele of *Lepr exon 1* in gonadotropes). Thus, the phenotype could become more severe with the next generation and we may be limited to F2-generation litters for future analyses.

To summarize, in this introductory section, we presented evidence that leptin is vital to the reproductive system. We also presented evidence suggesting that gonadotrope LEPR may be vital for optimal fertility. This evidence sets the stage for our two hypotheses in which we integrate findings from studies of neuronal and pituitary leptin-target cells. The first hypothesis will focus on leptin’s regulation of fertility *via* the gonadotrope, specifically regarding how the cyclic production of GnRHR proteins might provide a critical checkpoint for metabolic signaling. The second hypothesis will integrate the findings in the literature with those from our studies. In this hypothesis, we propose that leptin’s metabolic signaling involves multiple molecular gateways and checkpoints that can permit, delay, or stop reproduction.

## Hypothesis 1: Cyclic Regulation of Pituitary GnRHR Levels Sets up a Target Metabolic Checkpoint for Control of the Reproductive Axis

Pituitary gonadotropes in females are a fascinating subset of pituitary cells that must be remodeled every cycle to support a preovulatory LH surge and a postovulatory rise in FSH (109). Depending on the gonadotrope gene marker being detected and the stage of the cycle, these heterogeneous cells represent at least 15% of the pituitary population. Our studies over the past 42 years have shown that precise accounting of the gonadotrope population is complicated by their dynamic remodeling, such that they can be difficult to identify or detect when a marker gene product has been downregulated or secreted. At least two gonadotrope markers must be detected to identify the entire population, especially during periods of low gonadotropin storage (estrus or metestrus).

**Figure 2:**
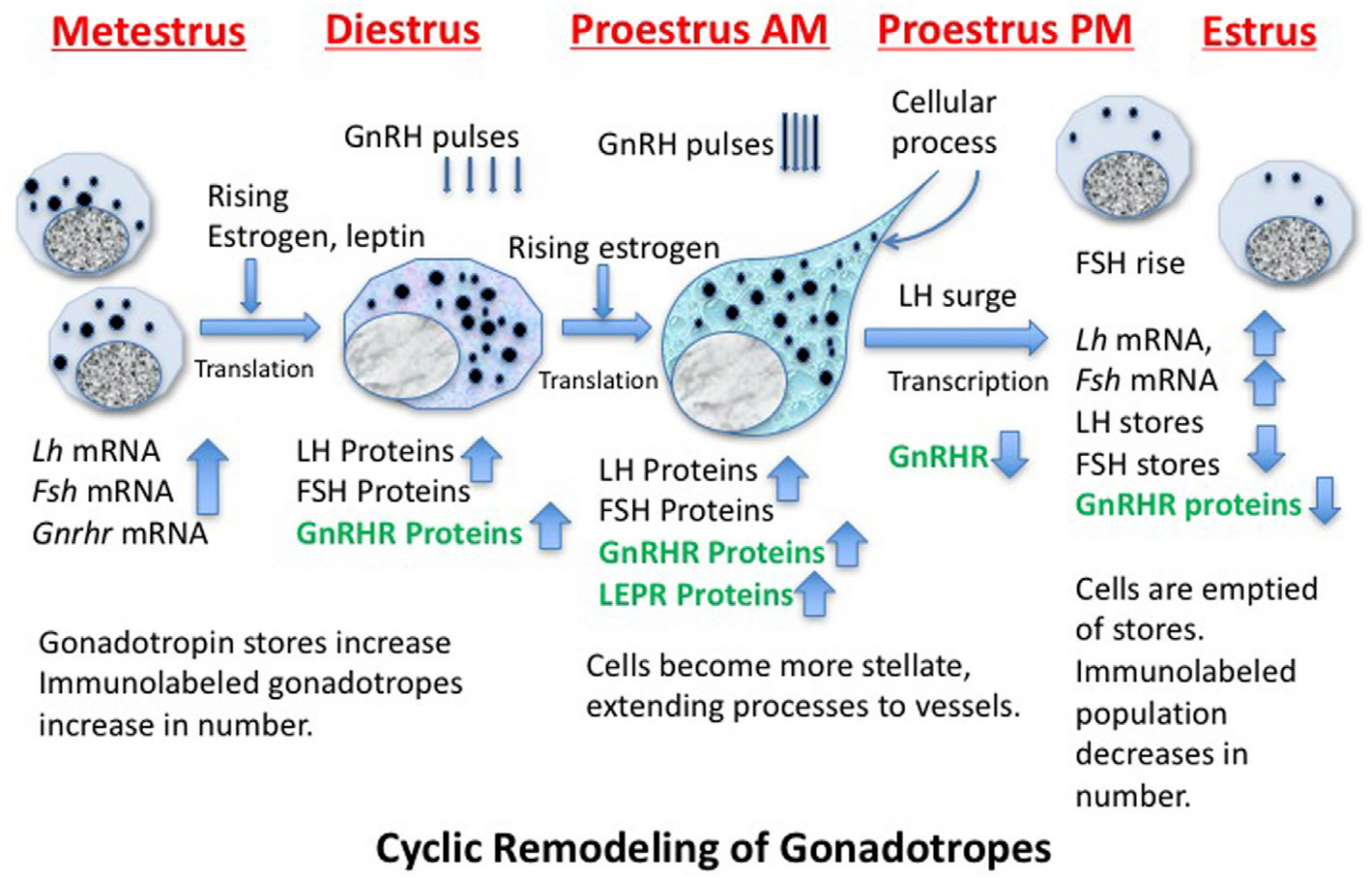
Cartoon showing stages of remodeling in the population of gonadotropes destined to support the luteinizing hormone (LH) surge secretory activity during proestrous and early estrus. The activity is driven by GnRH pulses, which increase in frequency and amplitude before the LH surge. The timing of the production of critical gene products is noted and those in green are known to be leptin targets.

Identification of the structural and molecular mechanisms behind the remodeling of gonadotropes and the regulators that drive these changes has been the subject of decades of investigative studies (109–116). Figure 2 shows a cartoon depicting some of the molecular and cytological changes that occur during the remodeling process that produces an actively secreting gonadotrope. Included in this population would presumably be any progenitor cell that contributes to the secreting, GnRH-responsive gonadotrope population, such as the somatogonadotrope (117).

Thanks to cytoskeletal remodeling, gonadotropes become more structurally elongated and stellate during diestrus and proestrus before the LH surge (109, 114, 118, 119), sending processes to blood vessels to facilitate surge-level secretion. Because they have actively secreted their stores during the LH surge and FSH rise, gonadotropin storage is significantly reduced on the morning of estrus (after the surge), reducing numbers of detectable gonadotropes (109, 110, 114, 120). Gonadotropes also increase their content of LEPRs during proestrus (33).

Figure 2 shows that, early in the cycle (metestrus), the cells destined to support the estrous rise in FSH and proestrous LH surge begin to produce gonadotropin and *Gnrhr* mRNA, which is followed by translation of these proteins during diestrus (121–123). The transcription of *Gnrhr* mRNA is under the control of GnRH pulses and rising levels of estrogen from the ovarian follicles (which had been stimulated by FSH early in estrus).More rapid pulses of GnRH in proestrus will facilitate the actual LH surge.

A critical step in this gonadotrope remodeling is the increase in GnRHR proteins. The changes in GnRHR depicted in Figure 2 were first reported by early radioreceptor assays, which detected the timing of the cyclic increase in GnRHR (121, 123) in rodents. The reports showed that gonadotropes undergo an increase in numbers of GnRHR early in diestrus I (metestrus) to reach a peak in late diestrus or on the morning of proestrus. Just before the LH surge, GnRHR numbers fall precipitously to remain low throughout the remaining stages of the cycle. This renders the gonadotrope population relatively quiescent during the postovulatory period of the cycle. There are LH and FSH pulses during this quiescent period, but they are of lower frequency and amplitude than those seen during mid-cycle surge activity.

The complex mechanisms controlling the increase in GnRHR clearly precede the remodeling needed to increase stores of gonadotropins needed for the surge activity, and it is not surprising that this initial process is regulated by pulses of GnRH itself (109). We have observed that the increase in GnRHR reflects an increase in the percentages of living gonadotropes that bind a biotinylated analog of GnRH (111). Collectively, these changes culminate in an increased population of responsive gonadotropes, which could then respond in synchrony to the higher GnRH pulse amplitude and frequency seen at mid-cycle.

The foregoing review of gonadotrope remodeling sets the stage for Hypothesis 1, which states that the cyclic changes in pituitary GnRHR expression create a mechanism by which the gonadotropes are activated only when environmental conditions are optimal. This mechanism would constitute an ideal checkpoint for metabolic regulation by leptin.

**Figure 3:**
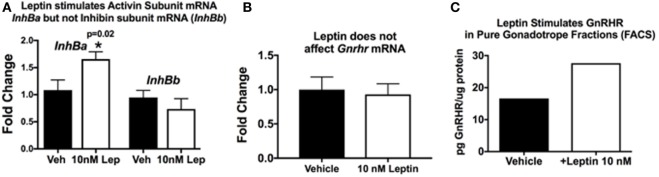
Pituitary responsiveness to leptin: activin and gonadotropin releasing hormone receptor (GnRHR). **(A,B)** Individual pituitaries were placed in 400 μL DMEM with protease inhibitor cocktail and ITS supplement (Sigma), with or without 10 nM recombinant mouse leptin (Sigma). The pituitaries were triturated two times with a 26 G needle and syringe. The pieces incubated for 3 h at 37°C, followed by mRNA extraction as previously described [(33)]. cDNAs were assayed by real-time PCR for the levels of activin/inhibin subunit **(A)** and *Gnrhr* **(B)** mRNA levels. Leptin stimulates an increase in pituitary activin subunit mRNA (*InhBa*) level and does not have an effect on inhibin (*InhBb*) or *Gnrhr* mRNA levels. **(C)** In a separate set of experiments, we used fluorescence-activated cell sorting (FACS) to purify gonadotropes from control females bearing *Lh-cre* and floxed alleles of *tdTomato-eGFP* as described previously (126). The Cre-recombinase ablates the *tdTomato* leaving *eGFP* to be expressed in membranes of gonadotropes. Our analysis showed that the eGFP fraction was 98–99% gonadotropes by immunolabeling for LH and follicle-stimulating hormone (FSH). In this experiment, a female gonadotrope fraction containing 66,830 cells was split and half of the cells were stimulated for 3 h with 10 nM leptin. Protein extracts of these cells were assayed by EIA for GnRHR (127). **(C)** GnRHR proteins were higher in the gonadotropes stimulated with leptin (from 16.67 µg GnRHR/mg protein to 27.6 µg/mg protein). The amount of protein in the extracts did not allow for multiple samples and, therefore, there are no error bars. More details about the GnRHR assays are reported in Akhter et al., (33). Details about the FACS protocol can be found in Odle et al. (126, 127).

This hypothesis originated when we discovered that GnRHR proteins and activin subunit mRNA levels were reduced in pituitaries lacking LEPRs in gonadotropes. Our studies of mice with LEPR ablated in gonadotropes discovered that both of these gene products were reduced (33). More recent studies determined if GnRHR and activin were direct targets of leptin. We assayed mRNA extracts from pituitary pieces that were stimulated for 3 h with 10 nM leptin. Methods describing our approach to leptin stimulation are detailed in previous studies (33, 52, 124, 125). Pituitary pieces or cells in 24-h culture are exposed to leptin for 3 h at 37°C, and then extracts of proteins and mRNA are produced, as described (126). Methods describing our RT-PCR assays are found in the legend to Figure 3 and in Ref. (33). Figure 3A shows leptin stimulation of pituitary activin (but not inhibin) subunit mRNA levels (see reference (33) for information on primer sets). Similarly, leptin stimulation for 3 h does not affect *Gnrhr* mRNA levels (Figure 3B). This correlates well with our previous study showing that lack of LEPR in gonadotropes does not affect *Gnrhr* mRNA (33).

Our *in vitro* studies also show that leptin stimulates GnRHR proteins in a dose-dependent manner, with 10 nM resulting in the highest levels of GnRHR proteins or percentages of cells that bind biotinylated analogs of GnRH (127). This study was recently expanded to determine if the gonadotropes were the target cells. We used our established fluorescence-activated cell sorting-purification protocol (126) to separate gonadotropes by their eGFP fluorescence (with mice bearing *Lh*-cre and floxed tdTomato-eGFP). Freshly purified gonadotrope fractions (66,000 cells) were split. Half of the population was stimulated for 3 h with 10 nM leptin, and the remaining half received vehicle. Immunolabeling showed that the eGFP fractions were 98% gonadotropes. EIAs showed that the fraction contained most of the GnRHR, LH, and FSH, with other hormones assayed in the non-eGFP fraction. Protein extracts from the leptin- or vehicle-treated gonadotropes were assayed for GnRHR as described (33). Figure 3C shows that the leptin stimulation resulted in an increase in GnRHR protein levels in this population of pure gonadotropes, which agrees with recently published evidence (127). This is the first evidence for leptin’s direct stimulation of gonadotropes.

Thus, collectively, our studies of gonadotrope-*Lepr*-null mice and *in vitro* responses to leptin highlight the importance of gonadotrope leptin-target cells to the HPG axis and support our hypothesis that gonadotrope GnRHR represents a metabolic checkpoint. However, we have expanded this hypothesis to include a novel leptin-mediated post-transcriptional pathway to control translation of the *Gnrhr* mRNA. This expansion is based on the following evidence: (1) GnRHR protein levels are reduced in gonadotrope *Lepr*-null mutants, but *Gnrhr* mRNA levels are unchanged (33); (2) leptin does not directly stimulate *Gnrhr* mRNA levels (Figure 3B), and (3) leptin directly stimulates GnRHR proteins in a population of purified gonadotropes (Figure 3C) or mixed pituitary cultures (127).

We propose that the levels of *Gnrhr* mRNA are normal in our gonadotrope-*Lepr* exon 17-null mutants likely because LEPR was not ablated in the hypothalamus, allowing GnRH secretion and the regulation of transcription of *Gnrhr, Lh*, and *Fsh* mRNA (33, 128, 129). LH and FSH stores are also normal in these mutant gonadotropes (33). However, the diestrous gonadotropes did not appear to secrete normally, as reported by low serum LH and FSH levels (33). Whereas we can explain the fact that *Gnrhr* mRNA is normal, the mechanism underlying leptin’s permissive modulation of GnRHR protein synthesis is unknown. As a first hypothesis, we, therefore, propose that leptin may stimulate translation by alleviating repression of *Gnrhr* mRNA by mRNA regulatory proteins and/or miRNA.

miRNAs are small (~22 nucleotides), single-stranded RNAs that interact with target sequences within cellular mRNAs and exert translational repression. A significant role for leptin signaling in regulation of miRNA-mediated translational control has been observed in adipocytes and hepatic cells (130). In *ob/ob* mice, *miR-103* and *miR-107* levels are increased in the absence of leptin, contributing directly to insulin resistance (130). Furthermore, leptin signaling involves JAK–STAT pathways and precedent for pSTAT3-dependent downregulation of target miRNAs has been reported in breast cancer (131).

**Figure 4:**
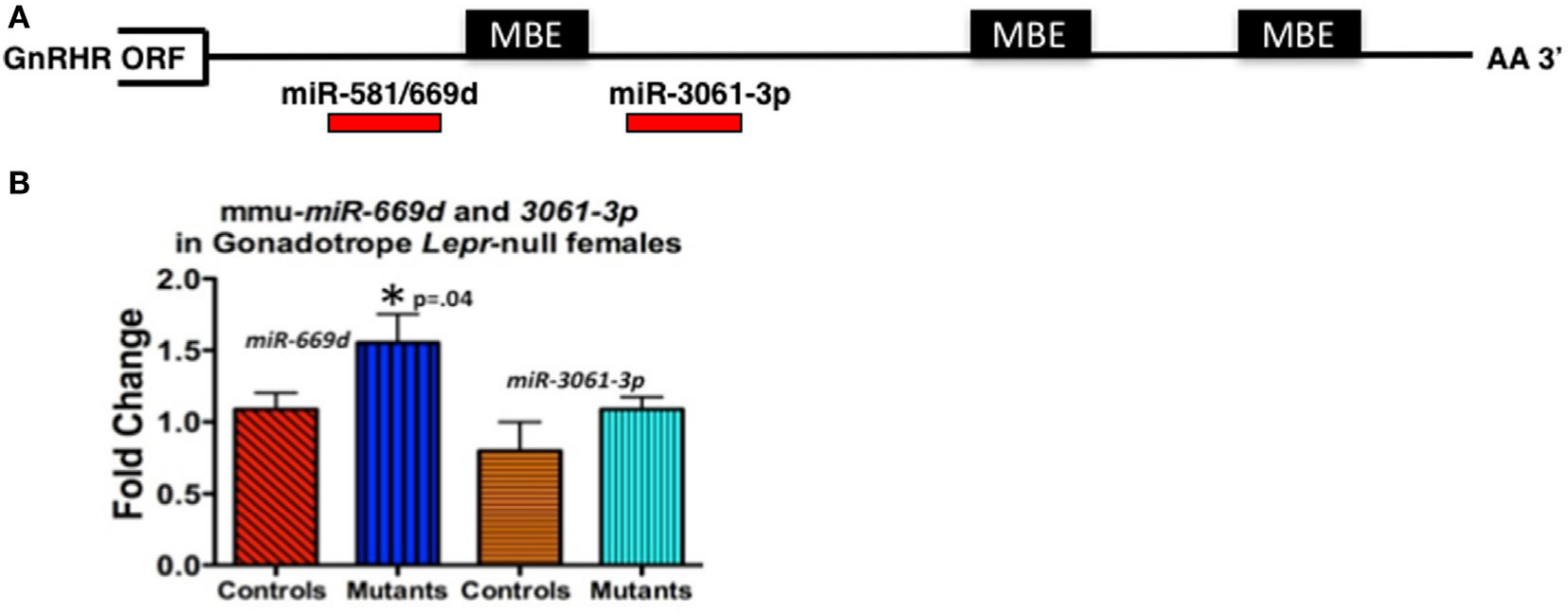
**(A)** Candidate regulatory elements within the murine *Gnrhr* 3′-untranslated region (UTR) include MBEs and at least two miRNA target sites. *In silico* analyses (TargetScan 7.1, ENSMUST00000031172.8) indicate three consensus MSI binding sites or elements (MBEs) and 16 miRNAs target sites (only 2 shown) within the murine *GnRHR* 3′UTR (182 nucleotides). The relative positions of the three MBEs and two miRNAs (*miR-581/669d* and *miR-3061-3p*) are shown schematically. The MBE closest to the open-reading frame (ORF) is flanked by sequences encoding *miR-581/669d* and *miR-3061-3p*. **(B)** *miR-581/669d* and *miR-3061-3p* levels in *Lepr-*null gonadotropes. Total RNA enriched for miRNA was isolated from whole pituitaries of control and gonadotrope-*LeprEx17-*null females using the Maxwell miRNA tissue kit (Promega, AS1470). We used the TaqMan MicroRNA Reverse Transcription Kit (Applied Biosystems, 4366596) with TaqMan small RNA assays to amplify our miRNAs of interest. We performed qRT-PCR using the TaqMan small RNA qPCR primers with the TaqMan Universal PCR Master Mix II (Applied Biosystems, 4440038) in triplicate. We used the protocol provided with the master mix and performed the experiment using a QuantStudio 12k Flex (Applied Biosystems) and the protocol provided with the master mix. Real-time PCR showed *miR-581/669d* elevated in the absence of leptin signaling, which is consistent with a role for this miRNA as a repressor of *Gnrhr* mRNA translation. *Significantly different from controls by Student’s *t*-test.

For our study, we initially wanted to determine which miRNAs might target the *Gnrhr* mRNA 3′-untranslated region (UTR). Our *in silico* analysis [Targetscan 7.1 (132)] revealed that the *Gnrhr* mRNA 3′-UTR (ENSMUST00000031172.8) contained 16 potential miRNA binding sites, including two that are also conserved in humans: *miR-581/669d* and *miR-3061-3p*. We began assays to detect differences, if any, in expression of candidate miRNAs. RT-PCR assays of whole pituitaries from control and gonadotrope *Lepr exon 17*–null diestrous females (*n* = 4–5 mice/group) determined that *miR-581/669d* was increased in the absence of leptin signals to gonadotropes, consistent with increased repression of *Gnrhr* mRNA translation in the mutants (Figure 4B). Detailed methods of our RT-PCR assays for miRNA are in the Figure legend of Figure 4. The specific role of *miR-581/669d* and the remaining 14 candidate miRNAs are currently being investigated. Complementary to this candidate approach, ongoing miRNA sequence analyses will provide an unbiased global analysis of pituitary miRNA expression related to loss of LEPR.

We also identified three consensus binding elements for the translational regulatory protein Musashi (MSI) (MBEs) in the 3′-UTR of murine *Gnrhr* mRNA (Figure 4A). The two vertebrate members of the MSI family, Musashi1 (MSI1) and Musashi2 (MSI2) are highly related, sequence-specific RNA binding proteins. MSI typically functions as a repressor of target mRNA translation and is specifically implicated in promoting stem/progenitor cell self-renewal where it functions to oppose translation of mRNAs encoding pro-differentiation factors and inhibitors of cell cycle progression under both physiological and pathological conditions (133). While the mouse pituitary is reported to express *Msi* mRNA (134), the function of MSI in the pituitary has not been determined. Ongoing studies show promise as they demonstrate binding by MSI to the *Gnrhr* mRNA 3′UTR and MSI repression of reporter expression driven by *Gnrhr* 3′UTR. We also have evidence that leptin reduces *Msi* mRNA (127).

Therefore, at this point, the evidence points to the hypothesis that leptin may play an important role in de-repressing *Gnrhr* mRNA during the critical period of cyclic upregulation of these vital receptors. We propose that MSI1 as well as at least one miRNA may be candidate *Gnrhr* mRNA regulators. Specifically, we propose that if nutrition and energy stores are normal, the mid-cycle leptin surge opposes MSI1-dependent mRNA repression, allowing the continued translation of *Gnrhr* during diestrus to reach optimal levels needed for a full LH surge. We propose that our mice that lack all isoforms of LEPR in gonadotropes (Table 1; Figure 1) may have not been able to produce sufficient GnRHR to mount an effective LH surge. Also, based on previous data (33) and Figure 3A, we propose that activin levels might be reduced, which would compromise FSH secretion and the development of the follicles. This first hypothesis will now be integrated into our second hypothesis about the overall mechanisms by which leptin signals metabolic information to the HPG axis.

## Hypothesis 2: Multiple Checkpoints are Required for Metabolic Signaling That Regulates the Reproductive Axis

As stated in the introduction, early studies of leptin regulation of reproduction have emphasized the hypothalamus as a primary target site for leptin and suggested that other sites might be less important or even redundant. The pioneering studies by McMinn et al. were the first to note the diversity in the LEPR-responsive neurons and the fact that all must be receptor deficient to cause infertility (8). Two studies selectively restored LEPR in the hypothalamus. The first of these studies reported that obesity, diabetes, and infertility in *Lepr*-null db/db mice could be rescued completely by re-introducing neuron-specific LEPR-B transgenes (82) to restore LEPR function selectively in the neurons. One of the drivers that introduced LEPR into the LEPR-null neurons was Synapsin (SYN-1). The selectivity of the SYN-1 driver was shown by expression in the brain; however, weak expression was also reported in the pituitary. This pituitary expression of SYN-1 was recently confirmed in Lβ-T2 gonadotropes and pituitary explants (135). Thus, based on the most recent evidence, we hypothesize that the Syn-1 driver may have introduced LEPR-B transgenes into both neuronal and anterior pituitary cells. Specifically, the expression of Synapsin in Lβ-T2 gonadotropes suggests that gonadotropes or their progenitor cells would have been among the restored leptin-target cells. Thus, fertility in these mice may have been restored by leptin-target neurons regulating GnRH and by leptin-target gonadotropes expressing GnRHR.

The second study by Donato et al. used Flp/FRT recombination approaches and a strain of mice carrying a neomycin cassette flanked by FRT sites targeted to the *Lepr* locus (50), which rendered the mutant mice globally LEPR-null. They selectively restored LEPR in the ventral premammillary (PMV) neurons of these mice by injecting an adeno-associated virus vector expressing Flp recombinase. The virus-restored mutant female mice showed evidence of pubertal development and cyclicity. In addition, five of the six females became pregnant although fertility was not optimal as four of these females did not carry the pups to term and the pups from the one female who delivered did not survive and died with no milk spots evident. These responses may also be due to the fact that the females remained morbidly obese. Thus, whereas the restoration of LEPR in the PMV clearly and selectively confirmed the importance of these neurons in the regulation of GnRH and the production of young, it appears that other leptin-target cells are vital to ensure that the progeny survive.

Based on our recent studies of *Lepr*-null gonadotropes (33), we hypothesize that the LEPR-null pituitaries in the study by Donato et al. expressed sufficient GnRHR on gonadotropes to go through puberty, cycle, and become pregnant. Because GnRH is an important stimulator of *Gnrhr* mRNA transcription [(128, 129) and Figure 2], restoration of LEPR in the PMV may have resulted in sufficient GnRH secretion to induce functional levels of GnRHR in gonadotropes. The observation that none of the litters survived, however, indicates that extra-PMV, pituitary, and ovarian LEPR-target cells are needed to support full reproductive competence. Also, the morbid obesity is a confounding factor. Detecting levels of gonadotropins, growth hormone, prolactin, estrogen, and progesterone may determine elements of the HPG axis that might have been most affected.

The importance of the working partnership between the hypothalamus and the pituitary is further elucidated in a recent study in which Cre-*LoxP* technology was used to restore only pituitary gonadotrope LEPR (101). As stated in the introduction, fertility was not restored in these animals presumably because LEPR-target neurons stimulating GnRH secretion remained deficient and unable to induce functional GnRHR signaling in gonadotropes (128, 129). This study provides another important clue to a role for leptin in gonadotropes, as they reported that FSH was elevated in this gonadotrope-specific LEPR model (101). As reported in our previous study (33), female mice bearing *Lepr*-null gonadotropes have reduced activin mRNA in the absence of leptin signals. We also reported reduced *Fsh* mRNA in these mutant animals. As activin stimulates FSH synthesis, we suggest that when LEPR was restored in pituitary gonadotropes, activin production may have been rescued (33). In the present report, we add evidence that leptin directly stimulates levels of activin mRNA (Figure 3A), which further supports this hypothesis. Also, recent studies of leptin actions in monkey pituitary cells show that 4 h of leptin stimulation *in vitro* results in elevated FSH secretion (55). It is interesting to note that leptin did not stimulate LH secretion *in vitro* in these female monkeys, which were reported to be of mixed cycles. We have shown that LEPRs in LH cells are maximal during the preovulatory period (33), and perhaps leptin’s effects on LH secretion are dependent on the stage of the menstrual cycle.

**Figure 5:**
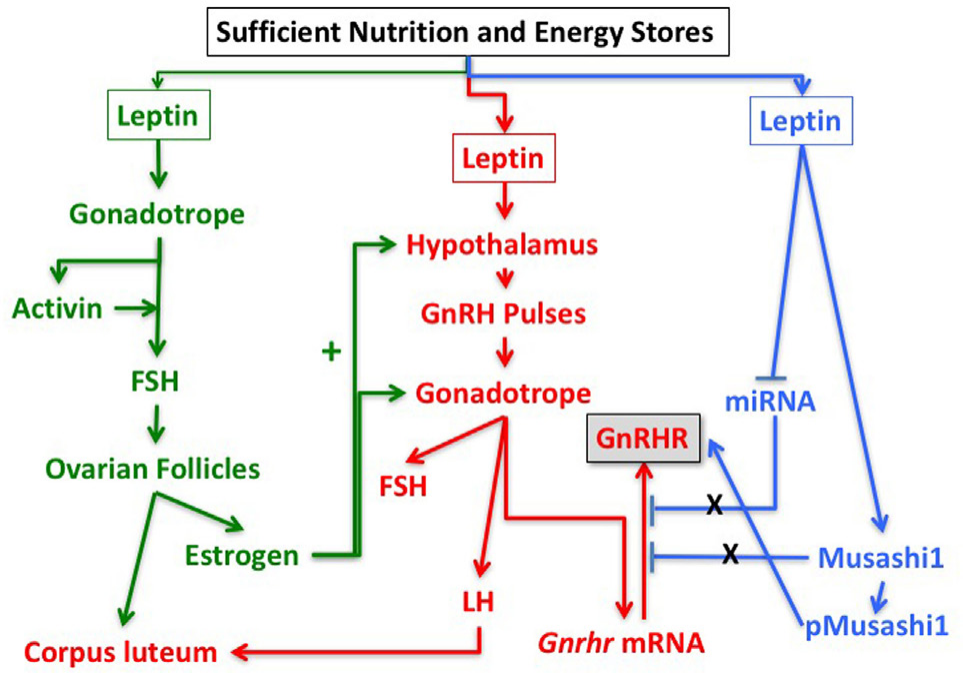
Pathways in the reproductive axis and the critical leptin targets that must be activated for reproductive competence. The pathway in green is proposed based on the evidence that leptin stimulates activin mRNA (Figure 2) and that activin mRNA is reduced in leptin receptor (LEPR)-null gonadotropes (33). It is also based on evidence that restoration of LEPR in LEPR-null gonadotropes results in elevated follicle-stimulating hormone (FSH) (101) as activin is important for FSH synthesis. We propose that this well-established FSH-driven pathway provides the stimulation to the follicle that results in a rise in estradiol needed early in the cycle to promote responses from GnRH neurons and gonadotropes. The red pathway is focused on the well-established circuitry in which leptin regulates LEPR-target neurons in the hypothalamus to stimulate GnRH neurons. It is based on evidence from key studies showing that restoration of LEPR in the PMV improves fertility (50, 82). The pathway shows that GnRH pulses are needed for transcription of *Gnrhr* mRNA as well as transcription and translation of LH and FSH. The blue pathway is based on our recent studies showing that mice lacking LEPR in gonadotropes are subfertile (33) or infertile (Figure 1; Table 1) and have significantly reduced gonadotropin releasing hormone receptor (GnRHR) proteins, but not mRNA levels (33). We hypothesize that leptin plays a direct post-transcriptional role in de-repressing mRNA translation by inhibiting miRNAs and/or MSI1, which then allows activation of *Gnrhr* mRNA translation.

Based on these findings and the studies described above, we hypothesize that leptin’s role in the permissive regulation of the reproductive cycle depends on timed events that involve multiple interactive target cells in the HPG axis. Figure 5 proposes a set of integrating pathways by which changing energy stores could allow leptin to signal metabolic information and permit, delay, or stop the next cycle. As shown in this figure, nutritional and fat level sufficiency will result in optimal leptin levels that in turn will signal target cells in the hypothalamus and pituitary gonadotropes. We hypothesize that leptin acts on hypothalamic and pituitary target cells to signal changing energy stores. The pathway designated in green shows how leptin may activate gonadotropes directly to effect transcription of activin subunits to raise local activin levels and stimulate synthesis of FSH. This would support the early estrous rise in FSH, which stimulates ovarian follicles to develop and secrete estradiol, which then exerts positive feedback on the hypothalamus and the pituitary. Estrogen-sensitive neuronal pathways stimulate GnRH neurons to increase secretion and pulse frequency. The pathway in red highlights the important role of leptin in stimulating the LEPR-sensitive neurons in the hypothalamus to ultimately regulate GnRH neurons. The red pathway also shows that GnRH pulses stimulate *Gnrhr* mRNA, as well as LH and FSH secretion. Most of the elements in the green and red pathways are well established, although the role of leptin in stimulating activin in the green pathway is relatively novel.

What is most novel is the hypothetical blue pathway. Based on our studies of gonadotrope *Lepr*-null mice, we propose that leptin sends a third signal directly to gonadotropes that de-represses the translation of *Gnrhr* mRNA. The timing of this gateway signal could be during the metestrous to diestrous increase in GnRHR proteins. Our studies of females that lack all isoforms of gonadotrope LEPR (Table 1; Figure 1) strongly emphasize the importance of this blue pathway for optimal reproductive success. As discussed for Hypothesis 1, we propose that *Gnrhr* mRNA translation may normally be inhibited by MSI1 and possibly miRNAs. Consequently, leptin signaling acts to de-repress the *Gnrhr* mRNA by blocking the inhibitory action of MSI1 and/or miRNA repressive activity. This ultimately would activate translation of *Gnrhr* mRNA and provide the full complement of receptors needed for a fully responsive gonadotrope population ready for the LH surge and estrous rise in FSH.

## Conclusion

Our two hypotheses reconcile and integrate findings from several studies of leptin-target cells. First, with the use of the Syn-1-driver, de Luca et al. (82) restored LEPR in both the brain and pituitary of *db/db* mice, which allowed multiple target cells seen in Figure 5 to function in partnership. Donato et al. (83) restored LEPR in the PMV of global LEPR-null mice, which stimulated GnRH to produce sufficient GnRHR and improve gonadotrope functions, although LEPR-target cells in the pituitary were still deficient and full reproduction (defined by the production of living pups) was not successful. As shown by Allen et al. (101) and Donato et al. (83), the system diagrammed in red and green pathways in Figure 5 will function only if leptin signaling to the brain is normal and only if there are GnRH pulses to stimulate the gonadotropes to make *Gnrhr* mRNA. However, as shown by our studies [Figure 1; Table 1; Ref. (33)], there must also be leptin input to gonadotropes for optimal levels of GnRHR proteins as well as responses to GnRHR for successful reproduction. Without that input, gonadotrope *Lepr exon 1-*null females failed to reproduce or had impaired fertility (Table 1).

Thus, the collective findings from the selective ablation or restoration of LEPR have highlighted the importance of leptin and LEPR to regulate function of the reproductive axis. Most importantly, they show that leptin’s permissive actions are operating in both the brain and the pituitary. These studies have further identified important pituitary signaling molecules activated directly or indirectly by leptin. Our hypotheses are integrated into the model in Figure 5 to indicate where each signal is needed and to highlight the fact that they act in partnership to optimize gonadotrope function. We also include a novel regulatory pathway that may involve control of MSI1 and/or miRNAs. Leptin regulation of these post-transcriptional pathways mediates the rapid de-repression and translation of *Gnrhr* mRNA, allowing for sufficient GnRHR to respond in synchrony and produce the LH surge. Subsequently, MSI1 and/or miRs would re-repress the *Gnrhr* mRNA late in the cycle, resulting in lower GnRHR levels and rendering the gonadotropes less responsive to GnRH. Continued studies are clearly needed to fully elucidate the targets and molecular pathways for leptin control of the HPG axis.

## Ethics Statement

This study was carried out under the guidelines of the Department of Lab Animal Medicine and the protocols were approved by the UAMS Animal Use and Care Committee.

## Author Note

These authors MM, AM and GC are all designated as Senior Authors on this manuscript as they supervised different elements of the studies that led to the manuscript.

## Author Contributions

AO performed the analysis of the estrous cycles in the gonadotrope LEPR-null females, all experiments involving leptin stimulation of gonadotropes and FACS separation of gonadotropes, all qPCR assays for mRNA and miRNAs. She also helped with the literature review and the development of the hypotheses. NA and MS performed the cytochemical labeling studies that are cited in this work showing leptin stimulation of GnRHR. MA-J helped with cyclicity studies and cytochemistry cited in the paper. HB, MC, and MM worked on studies of MSI binding that are cited in this paper. AM did the *in silico* analysis of the GnRHR 3′-UTR and, with MM, designed experiments to test MSI binding. GC set up and monitored all breeding studies and wrote the initial drafts of the manuscript. All authors helped with the development of the hypotheses and the editing. MM, AM and GC are Co-Senior authors on this study working as equal partners in the development of the hypotheses and monitoring the final writing and editing.

## Conflict of Interest Statement

The authors declare that the research was conducted in the absence of any commercial or financial relationships that could be construed as a potential conflict of interest.

## References

[1] Ahima RS Dushay J Flier SN Prabakaran D Flier JS. Leptin accelerates the onset of puberty in normal female mice. J Clin Invest (1997) 99:391–5. 10.1172/JCI119172 9022071 PMC507811

[2] Ahima RS Prabakaran D Mantzoros C Qu D Lowell B Maratos-Flier E Role of leptin in the neuroendocrine response to fasting. Nature (1996) 382:250–2. 10.1038/382250a0 8717038

[3] Barash IA Cheung CC Weigle DS Ren H Kabigting EB Kuijper JL Leptin is a metabolic signal to the reproductive system. Endocrinology (1996) 137:3144–7. 10.1210/endo.137.7.8770941 8770941

[4] Finn PD Cunningham MJ Pau KY Spies HG Clifton DK Steiner RA. The stimulatory effect of leptin on the neuroendocrine reproductive axis of the monkey. Endocrinology (1998) 139:4652–62. 10.1210/endo.139.11.6297 9794477

[5] Ishii S Shibasaki T Murakami T Shima K Wakabayashi I. Response of leptin mRNA to 24-h food deprivation and refeeding is influenced by age in rats. Regul Pept (2000) 92:45–50. 10.1016/S0167-0115(00)00148-8 11024564

[6] Mann DR Plant TM. Leptin and pubertal development. Semin Reprod Med (2002) 20:93–102. 10.1055/s-2002-32500 12087494

[7] Urbanski HF. Leptin and puberty. Trends Endocrinol Metab (2001) 12:428–9. 10.1016/S1043-2760(01)00505-7 11701332

[8] McMinn JE Liu SM Liu H Dragatsis I Dietrich P Ludwig T Neuronal deletion of Lepr elicits diabesity in mice without affecting cold tolerance or fertility. Am J Physiol Endocrinol Metab (2005) 289:E403–11. 10.1152/ajpendo.00535.2004 15870101

[9] Considine RV Sinha MK Heiman ML Kriauciunas A Stephens TW Nyce MR Serum immunoreactive-leptin concentrations in normal-weight and obese humans. N Engl J Med (1996) 334:292–5. 10.1056/NEJM199602013340503 8532024

[10] Maffei M Halaas J Ravussin E Pratley RE Lee GH Zhang Y Leptin levels in human and rodent: measurement of plasma leptin and ob RNA in obese and weight-reduced subjects. Nat Med (1995) 1:1155–61. 10.1038/nm1195-1155 7584987

[11] Zhang Y Proenca R Maffei M Barone M Leopold L Friedman JM. Positional cloning of the mouse obese gene and its human homologue. Nature (1994) 372:425–32. 10.1038/372425a0 7984236

[12] Ahima RS Saper CB Flier JS Elmquist JK. Leptin regulation of neuroendocrine systems. Front Neuroendocrinol (2000) 21:263–307. 10.1006/frne.2000.0197 10882542

[13] Casanueva F Dieguez C Interactions between body composition, leptin and growth hormone status. In: Shalet SM, editor. Growth Hormone in Adults. (Vol. 12), London: Bailliere Tindall (1998). p. 297–314. 10.1016/s0950-351x(98)80024-4 10083898

[14] Casanueva FF Dieguez C. Neuroendocrine regulation and actions of leptin. Front Neuroendocrinol (1999) 20:317–63. 10.1006/frne.1999.0187 10569281

[15] Castellano JM Bentsen AH Sanchez-Garrido MA Ruiz-Pino F Romero M Garcia-Galiano D Early metabolic programming of puberty onset: impact of changes in postnatal feeding and rearing conditions on the timing of puberty and development of the hypothalamic kisspeptin system. Endocrinology (2011) 152:3396–408. 10.1210/en.2010-1415 21712362

[16] Chan JL Mantzoros CS. Role of leptin in energy-deprivation states: normal human physiology and clinical implications for hypothalamic amenorrhoea and anorexia nervosa. Lancet (2005) 366:74–85. 10.1016/S0140-6736(05)66830-4 15993236

[17] Flier JS Clinical review 94: what’s in a name? In search of leptin’s physiologic role. J Clin Endocrinol Metab (1998) 83:1407–13. 10.1210/jcem.83.5.4779 9589630

[18] Kopp W Blum WF von Prittwitz S Ziegler A Lubbert H Emons G Low leptin levels predict amenorrhea in underweight and eating disordered females. Mol Psychiatry (1997) 2:335–40. 10.1038/sj.mp.4000287 9246675

[19] Laughlin GA Yen SS. Hypoleptinemia in women athletes: absence of a diurnal rhythm with amenorrhea. J Clin Endocrinol Metab (1997) 82:318–21. 10.1210/jcem.82.1.3840 8989281

[20] Clement K Vaisse C Lahlou N Cabrol S Pelloux V Cassuto D A mutation in the human leptin receptor gene causes obesity and pituitary dysfunction. Nature (1998) 392:398–401. 10.1038/32911 9537324

[21] Kiess W Blum WF Aubert ML Leptin, puberty and reproductive function: lessons from animal studies and observations in humans. Eur J Endocrinol (1998) 138:26–9. 10.1530/eje.0.1380026 9461310

[22] Miller KK Parulekar MS Schoenfeld E Anderson E Hubbard J Klibanski A Decreased leptin levels in normal weight women with hypothalamic amenorrhea: the effects of body composition and nutritional intake. J Clin Endocrinol Metab (1998) 83:2309–12. 10.1210/jcem.83.7.4975 9661600

[23] Cunningham MJ Clifton DK Steiner RA. Leptin’s actions on the reproductive axis: perspectives and mechanisms. Biol Reprod (1999) 60:216–22. 10.1095/biolreprod60.2.216 9915984

[24] Thong FS Graham TE. Leptin and reproduction: is it a critical link between adipose tissue, nutrition, and reproduction? Can J Appl Physiol (1999) 24:317–36. 10.1139/h99-025 10470449

[25] Ahima RS Flier JS. Leptin. Annu Rev Physiol (2000) 62:413–37. 10.1146/annurev.physiol.62.1.413 10845097

[26] Caprio M Fabbrini E Isidori AM Aversa A Fabbri A. Leptin in reproduction. Trends Endocrinol Metab (2001) 12:65–72. 10.1016/S1043-2760(00)00352-0 11167124

[27] Chan JL Mantzoros CS. Leptin and the hypothalamic-pituitary regulation of the gonadotropin-gonadal axis. Pituitary (2001) 4:87–92. 10.1023/A:1012947113197 11824513

[28] Popovic V Damjanovic S Dieguez C Casanueva FF Leptin and the pituitary. Pituitary (2001) 4:7–14. 10.1023/A:1012938308654 11824510

[29] Pralong FP Gaillard RC. Neuroendocrine effects of leptin. Pituitary (2001) 4:25–32. 10.1023/A:1012930609563 11824505

[30] Spicer LJ. Leptin: a possible metabolic signal affecting reproduction. Domest Anim Endocrinol (2001) 21:251–70. 10.1016/S0739-7240(01)00120-5 11872320

[31] Bluher S Mantzoros CS. Leptin in reproduction. Curr Opin Endocrinol Diabetes Obes (2007) 14:458–64. 10.1097/MED.0b013e3282f1cfdc 17982352

[32] Akhter N Crane C Childs GV Pituitary leptin-A paracrine regulator of gonadotropes: a review. Open Neuroendocrinol J (2011) 4:25–42. 10.2174/1876528901104010025

[33] Akhter N CarlLee T Syed MM Odle AK Cozart MA Haney AC Selective deletion of leptin receptors in gonadotropes reveals activin and GnRH-binding sites as leptin targets in support of fertility. Endocrinology (2014) 155:4027–42. 10.1210/en.2014-1132 25057790 PMC4164926

[34] Montague CT Farooqi IS Whitehead JP Soos MA Rau H Wareham NJ Congenital leptin deficiency is associated with severe early-onset obesity in humans. Nature (1997) 387:903–8. 10.1038/43185 9202122

[35] Strobel A Issad T Camoin L Ozata M Strosberg AD A leptin missense mutation associated with hypogonadism and morbid obesity. Nat Genet (1998) 18:213–5. 10.1038/ng0398-213 9500540

[36] Kaufman BA Warren MP Dominguez JE Wang J Heymsfield SB Pierson RN. Bone density and amenorrhea in ballet dancers are related to a decreased resting metabolic rate and lower leptin levels. J Clin Endocrinol Metab (2002) 87:2777–83. 10.1210/jcem.87.6.8565 12050250

[37] Thong FS McLean C Graham TE. Plasma leptin in female athletes: relationship with body fat, reproductive, nutritional, and endocrine factors. J Appl Physiol (2000) 88:2037–44. 10846016 10.1152/jappl.2000.88.6.2037

[38] Warren MP Voussoughian F Geer EB Hyle EP Adberg CL Ramos RH. Functional hypothalamic amenorrhea: hypoleptinemia and disordered eating. J Clin Endocrinol Metab (1999) 84:873–7. 10.1210/jcem.84.3.5551 10084564

[39] Welt CK Will leptin become the treatment of choice for functional hypothalamic amenorrhea? Nat Clin Pract Endocrinol Metab (2007) 3:556–7. 10.1038/ncpendmet0561 17593916

[40] Welt CK Chan JL Bullen J Murphy R Smith P DePaoli AM Recombinant human leptin in women with hypothalamic amenorrhea. N Engl J Med (2004) 351:987–97. 10.1056/NEJMoa040388 15342807

[41] Licinio J Negrao AB Mantzoros C Kaklamani V Wong ML Bongiorno PB Synchronicity of frequently sampled, 24-h concentrations of circulating leptin, luteinizing hormone, and estradiol in healthy women. Proc Natl Acad Sci U S A (1998) 95:2541–6. 10.1073/pnas.95.5.2541 9482922 PMC19406

[42] Farooqi IS Jebb SA Langmack G Lawrence E Cheetham CH Prentice AM Effects of recombinant leptin therapy in a child with congenital leptin deficiency. N Engl J Med (1999) 341:879–84. 10.1056/NEJM199909163411204 10486419

[43] Chan JL Heist K DePaoli AM Veldhuis JD Mantzoros CS. The role of falling leptin levels in the neuroendocrine and metabolic adaptation to short-term starvation in healthy men. J Clin Invest (2003) 111:1409–21. 10.1172/JCI200317490 12727933 PMC154448

[44] Cagampang FR Maeda K Yokoyama A Ota K. Effect of food deprivation on the pulsatile LH release in the cycling and ovariectomized female rat. Horm Metab Res (1990) 22:269–72. 10.1055/s-2007-1004900 2347540

[45] Cameron JL Nosbisch C. Suppression of pulsatile luteinizing hormone and testosterone secretion during short term food restriction in the adult male rhesus monkey (*Macaca mulatta*). Endocrinology (1991) 128:1532–40. 10.1210/endo-128-3-1532 1999171

[46] Cameron JL Weltzin TE McConaha C Helmreich DL Kaye WH. Slowing of pulsatile luteinizing hormone secretion in men after forty-eight hours of fasting. J Clin Endocrinol Metab (1991) 73:35–41. 10.1210/jcem-73-1-35 1904451

[47] Weigle DS Duell PB Connor WE Steiner RA Soules MR Kuijper JL. Effect of fasting, refeeding, and dietary fat restriction on plasma leptin levels. J Clin Endocrinol Metab (1997) 82:561–5. 10.1210/jc.82.2.561 9024254

[48] Parfitt DB Church KR Cameron JL. Restoration of pulsatile luteinizing hormone secretion after fasting in rhesus monkeys (*Macaca mulatta*): dependence on size of the refeed meal. Endocrinology (1991) 129:749–56. 10.1210/endo-129-2-749 1855472

[49] Carro E Pinilla L Seoane LM Considine RV Aguilar E Casanueva FF Influence of endogenous leptin tone on the estrous cycle and luteinizing hormone pulsatility in female rats. Neuroendocrinology (1997) 66:375–7. 10.1159/000127262 9430442

[50] Donato J Jr Silva RJ Sita LV Lee S Lee C Lacchini S The ventral premammillary nucleus links fasting-induced changes in leptin levels and coordinated luteinizing hormone secretion. J Neurosci (2009) 29:5240–50. 10.1523/JNEUROSCI.0405-09.2009 19386920 PMC2696192

[51] Gonzalez LC Pinilla L Tena-Sempere M Aguilar E. Leptin(116–130) stimulates prolactin and luteinizing hormone secretion in fasted adult male rats. Neuroendocrinology (1999) 70:213–20. 10.1159/000054479 10516485

[52] Crane C Akhter N Johnson BW Iruthayanathan M Syed F Kudo A Fasting and glucose effects on pituitary leptin expression: is leptin a local signal for nutrient status? J Histochem Cytochem (2007) 55:1059–74. 10.1369/jhc.7A7214.2007 17595338 PMC2085236

[53] Chehab FF Mounzih K Lu R Lim ME. Early onset of reproductive function in normal female mice treated with leptin. Science (1997) 275:88–90. 10.1126/science.275.5296.88 8974400

[54] Chehab FF Lim ME Lu R. Correction of the sterility defect in homozygous obese female mice by treatment with the human recombinant leptin. Nat Genet (1996) 12:318–20. 10.1038/ng0396-318 8589726

[55] Sarmento-Cabral A Peinado JR Halliday LC Malagon MM Castano JP Kineman RD Adipokines (leptin, adiponectin, resistin) differentially regulate all hormonal cell types in primary anterior pituitary cell cultures from two primate species. Sci Rep (2017) 7:43537. 10.1038/srep43537 28349931 PMC5640086

[56] Cheung CC Thornton JE Kuijper JL Weigle DS Clifton DK Steiner RA. Leptin is a metabolic gate for the onset of puberty in the female rat. Endocrinology (1997) 138:855–8. 10.1210/endo.138.2.5054 9003028

[57] Bronson FH. Food-restricted, prepubertal, female rats: rapid recovery of luteinizing hormone pulsing with excess food, and full recovery of pubertal development with gonadotropin-releasing hormone. Endocrinology (1986) 118:2483–7. 10.1210/endo-118-6-2483 3516663

[58] Bronson FH. Puberty in female mice is not associated with increases in either body fat or leptin. Endocrinology (2001) 142:4758–61. 10.1210/endo.142.11.8495 11606441

[59] Cheung CC Thornton JE Nurani SD Clifton DK Steiner RA. A reassessment of leptin’s role in triggering the onset of puberty in the rat and mouse. Neuroendocrinology (2001) 74:12–21. 10.1159/000054666 11435754

[60] Mann DR Akinbami MA Gould KG Castracane VD. Leptin and thyroxine during sexual development in male monkeys: effect of neonatal gonadotropin-releasing hormone antagonist treatment and delayed puberty on the developmental pattern of leptin and thyroxine secretion. Eur J Endocrinol (2002) 146:891–8. 10.1530/eje.0.1460891 12039711

[61] Mann DR Bhat GK Ramaswamy S Stah CD Plant TM. Regulation of circulating leptin and its soluble receptor during pubertal development in the male rhesus monkey (*Macaca mulatta*). Endocrine (2007) 31:125–9. 10.1007/s12020-007-0020-0 17873322

[62] Plant TM Durrant AR. Circulating leptin does not appear to provide a signal for triggering the initiation of puberty in the male rhesus monkey (*Macaca mulatta*). Endocrinology (1997) 138:4505–8. 10.1210/endo.138.10.5574 9322973

[63] Urbanski HF Pau KY. A biphasic developmental pattern of circulating leptin in the male rhesus macaque (*Macaca mulatta*). Endocrinology (1998) 139:2284–6. 10.1210/endo.139.5.5962 9564835

[64] Reitman ML Bi S Marcus-Samuels B Gavrilova O Leptin and its role in pregnancy and fetal development – an overview. Biochem Soc Trans (2001) 29:68–72. 10.1042/bst0290068 11356129

[65] Ahima RS Prabakaran D Flier JS. Postnatal leptin surge and regulation of circadian rhythm of leptin by feeding. Implications for energy homeostasis and neuroendocrine function. J Clin Invest (1998) 101:1020–7. 10.1172/JCI1176 9486972 PMC508653

[66] Devaskar SU Ollesch C Rajakumar RA Rajakumar PA. Developmental changes in ob gene expression and circulating leptin peptide concentrations. Biochem Biophys Res Commun (1997) 238:44–7. 10.1006/bbrc.1997.7237 9299448

[67] Zamorano PL Mahesh VB De Sevilla LM Chorich LP Bhat GK Brann DW. Expression and localization of the leptin receptor in endocrine and neuroendocrine tissues of the rat. Neuroendocrinology (1997) 65:223–8. 10.1159/000127276 9088004

[68] Lebrethon MC Vandersmissen E Gerard A Parent AS Bourguignon JP. Cocaine and amphetamine-regulated-transcript peptide mediation of leptin stimulatory effect on the rat gonadotropin-releasing hormone pulse generator in vitro. J Neuroendocrinol (2000) 12:383–5. 10.1046/j.1365-2826.2000.00497.x 10792575

[69] Lebrethon MC Vandersmissen E Gerard A Parent AS Junien JL Bourguignon JP. In vitro stimulation of the prepubertal rat gonadotropin-releasing hormone pulse generator by leptin and neuropeptide Y through distinct mechanisms. Endocrinology (2000) 141:1464–9. 10.1210/endo.141.4.7432 10746651

[70] Nagatani S Guthikonda P Thompson RC Tsukamura H Maeda KI Foster DL. Evidence for GnRH regulation by leptin: leptin administration prevents reduced pulsatile LH secretion during fasting. Neuroendocrinology (1998) 67:370–6. 10.1159/000054335 9662716

[71] Watanobe H. Leptin directly acts within the hypothalamus to stimulate gonadotropin-releasing hormone secretion in vivo in rats. J Physiol (2002) 545:255–68. 10.1113/jphysiol.2002.023895 12433965 PMC2290656

[72] Elias CF Lee C Kelly J Aschkenasi C Ahima RS Couceyro PR Leptin activates hypothalamic CART neurons projecting to the spinal cord. Neuron (1998) 21:1375–85. 10.1016/S0896-6273(00)80656-X 9883730

[73] Korner J Savontaus E Chua SC Jr Leibel RL Wardlaw SL. Leptin regulation of Agrp and Npy mRNA in the rat hypothalamus. J Neuroendocrinol (2001) 13:959–66. 10.1046/j.1365-2826.2001.00716.x 11737554

[74] Balthasar N Coppari R McMinn J Liu SM Lee CE Tang V Leptin receptor signaling in POMC neurons is required for normal body weight homeostasis. Neuron (2004) 42:983–91. 10.1016/j.neuron.2004.06.004 15207242

[75] Bouret SG Draper SJ Simerly RB. Trophic action of leptin on hypothalamic neurons that regulate feeding. Science (2004) 304:108–10. 10.1126/science.1095004 15064420

[76] Elmquist JK Flier JS Neuroscience. The fat-brain axis enters a new dimension. Science (2004) 304:63–4. 10.1126/science.1096746 15064411

[77] Morrison CD Morton GJ Niswender KD Gelling RW Schwartz MW. Leptin inhibits hypothalamic Npy and Agrp gene expression via a mechanism that requires phosphatidylinositol 3-OH-kinase signaling. Am J Physiol Endocrinol Metab (2005) 289:E1051–7. 10.1152/ajpendo.00094.2005 16046456

[78] Lehman MN Coolen LM Goodman RL. Minireview: kisspeptin/neurokinin B/dynorphin (KNDy) cells of the arcuate nucleus: a central node in the control of gonadotropin-releasing hormone secretion. Endocrinology (2010) 151:3479–89. 10.1210/en.2010-0022 20501670 PMC2940527

[79] Lehman MN Merkley CM Coolen LM Goodman RL. Anatomy of the kisspeptin neural network in mammals. Brain Res (2010) 1364:90–102. 10.1016/j.brainres.2010.09.020 20858464 PMC2992597

[80] True C Grove KL Smith MS. Beyond leptin: emerging candidates for the integration of metabolic and reproductive function during negative energy balance. Front Endocrinol (2011) 2:53. 10.3389/fendo.2011.00053 22645510 PMC3355832

[81] True C Kirigiti MA Kievit P Grove KL Smith MS. Leptin is not the critical signal for kisspeptin or luteinising hormone restoration during exit from negative energy balance. J Neuroendocrinol (2011) 23:1099–112. 10.1111/j.1365-2826.2011.02144.x 21518032 PMC3646420

[82] de Luca C Kowalski TJ Zhang Y Elmquist JK Lee C Kilimann MW Complete rescue of obesity, diabetes, and infertility in db/db mice by neuron-specific LEPR-B transgenes. J Clin Invest (2005) 115:3484–93. 10.1172/JCI24059 16284652 PMC1280964

[83] Donato J Jr Cravo RM Frazao R Gautron L Scott MM Lachey J Leptin’s effect on puberty in mice is relayed by the ventral premammillary nucleus and does not require signaling in Kiss1 neurons. J Clin Invest (2011) 121:355–68. 10.1172/JCI45106 21183787 PMC3007164

[84] Elias CF. Leptin action in pubertal development: recent advances and unanswered questions. Trends Endocrinol Metab (2012) 23:9–15. 10.1016/j.tem.2011.09.002 21978495 PMC3251729

[85] Elias CF Purohit D. Leptin signaling and circuits in puberty and fertility. Cell Mol Life Sci (2013) 70:841–62. 10.1007/s00018-012-1095-1 22851226 PMC3568469

[86] Iqbal J Kurose Y Canny B Clarke IJ. Effects of central infusion of ghrelin on food intake and plasma levels of growth hormone, luteinizing hormone, prolactin, and cortisol secretion in sheep. Endocrinology (2006) 147:510–9. 10.1210/en.2005-1048 16210361

[87] Iqbal J Pompolo S Considine RV Clarke IJ. Localization of leptin receptor-like immunoreactivity in the corticotropes, somatotropes, and gonadotropes in the ovine anterior pituitary. Endocrinology (2000) 141:1515–20. 10.1210/endo.141.4.7433 10746658

[88] Jin L Burguera BG Couce ME Scheithauer BW Lamsan J Eberhardt NL Leptin and leptin receptor expression in normal and neoplastic human pituitary: evidence of a regulatory role for leptin on pituitary cell proliferation. J Clin Endocrinol Metab (1999) 84:2903–11. 10.1210/jc.84.8.2903 10443698

[89] Jin L Zhang S Burguera BG Couce ME Osamura RY Kulig E Leptin and leptin receptor expression in rat and mouse pituitary cells. Endocrinology (2000) 141:333–9. 10.1210/endo.141.1.7260 10614655

[90] Lloyd RV Jin L Qian X Zhang S Scheithauer BW. Nitric oxide synthase in the human pituitary gland. Am J Pathol (1995) 146:86–94. 7531951 PMC1870770

[91] Lloyd RV Jin L Tsumanuma I Vidal S Kovacs K Horvath E Leptin and leptin receptor in anterior pituitary function. Pituitary (2001) 4:33–47. 10.1023/A:1012982626401 11824506

[92] Giusti M Bocca L Florio T Corsaro A Spaziante R Schettini G In vitro effect of human recombinant leptin and expression of leptin receptors on growth hormone-secreting human pituitary adenomas. Clin Endocrinol (Oxf) (2002) 57:449–55. 10.1046/j.1365-2265.2002.01612.x 12354126

[93] Sone M Osamura RY Leptin and the pituitary. Pituitary (2001) 4:15–23. 10.1023/A:1012978525492 11824504

[94] Yura S Ogawa Y Sagawa N Masuzaki H Itoh H Ebihara K Accelerated puberty and late-onset hypothalamic hypogonadism in female transgenic skinny mice overexpressing leptin. J Clin Invest (2000) 105:749–55. 10.1172/JCI8353 10727443 PMC377463

[95] De Biasi SN Apfelbaum LI Apfelbaum ME. In vitro effect of leptin on LH release by anterior pituitary glands from female rats at the time of spontaneous and steroid-induced LH surge. Eur J Endocrinol (2001) 145:659–65. 10.1530/eje.0.1450659 11720886

[96] Ogura K Irahara M Kiyokawa M Tezuka M Matsuzaki T Yasui T Effects of leptin on secretion of LH and FSH from primary cultured female rat pituitary cells. Eur J Endocrinol (2001) 144:653–8. 10.1530/eje.0.1440653 11375800

[97] Tezuka M Irahara M Ogura K Kiyokawa M Tamura T Matsuzaki T Effects of leptin on gonadotropin secretion in juvenile female rat pituitary cells. Eur J Endocrinol (2002) 146:261–6. 10.1530/eje.0.1460261 11834438

[98] Swerdloff RS Batt RA Bray GA. Reproductive hormonal function in the genetically obese (ob/ob) mouse. Endocrinology (1976) 98:1359–64. 10.1210/endo-98-6-1359 1278106

[99] Swerdloff RS Peterson M Vera A Batt RA Heber D Bray GA The hypothalamic-pituitary axis in genetically obese (ob/ob) mice: response to luteinizing hormone-releasing hormone. Endocrinology (1978) 103:542–7. 10.1210/endo-103-2-542 369840

[100] Yu WH Kimura M Walczewska A Karanth S McCann SM. Role of leptin in hypothalamic-pituitary function. Proc Natl Acad Sci U S A (1997) 94:1023–8. 10.1073/pnas.94.3.1023 9023376 PMC19633

[101] Allen SJ Garcia-Galiano D Borges BC Burger LL Boehm U Elias CF. Leptin receptor null mice with reexpression of LepR in GnRHR expressing cells display elevated FSH levels but remain in a prepubertal state. Am J Physiol Regul Integr Comp Physiol (2016) 310:R1258–66. 10.1152/ajpregu.00529.2015 27101301 PMC4935496

[102] Fortin J Boehm U Deng CX Treier M Bernard DJ. Follicle-stimulating hormone synthesis and fertility depend on SMAD4 and FOXL2. FASEB J (2014) 28:3396–410. 10.1096/fj.14-249532 24739304 PMC4101660

[103] Fortin J Boehm U Weinstein MB Graff JM Bernard DJ. Follicle-stimulating hormone synthesis and fertility are intact in mice lacking SMAD3 DNA binding activity and SMAD2 in gonadotrope cells. FASEB J (2014) 28:1474–85. 10.1096/fj.13-237818 24308975 PMC3929678

[104] Li Y Schang G Boehm U Deng CX Graff J Bernard DJ. SMAD3 regulates follicle-stimulating hormone synthesis by pituitary gonadotrope cells in vivo. J Biol Chem (2017) 292:2301–14. 10.1074/jbc.M116.759167 27994055 PMC5313102

[105] Cohen P Zhao C Cai X Montez JM Rohani SC Feinstein P Selective deletion of leptin receptor in neurons leads to obesity. J Clin Invest (2001) 108:1113–21. 10.1172/JCI200113914 11602618 PMC209535

[106] Allensworth-James M Odle AK Haney A Childs GV. Sex differences in somatotrope dependency on leptin receptors in young mice: ablation of LEPR causes severe growth hormone deficiency and abdominal obesity in males. Endocrinology (2015) 156:3253–64. 10.1210/EN.2015-1198 26168341 PMC4541611

[107] Akhter N Johnson BW Crane C Iruthayanathan M Zhou Y-H Kudo A Anterior pituitary leptin expression changes in different reproductive states: in vitro stimulation by gonadotropin-releasing hormone. J Histochem Cytochem (2007) 55:151–66. 10.1369/jhc.6A7072.2006 17046838 PMC1780073

[108] Charles MA Mortensen AH Potok MA Camper SA. Pitx2 deletion in pituitary gonadotropes is compatible with gonadal development, puberty, and fertility. Genesis (2008) 46:507–14. 10.1002/dvg.20398 18802953 PMC2923441

[109] Childs GV Gonadotropes and lactotropes. In: Neill J Knobil E, editors. Physiology of Reproduction. New York, NY: Elsevier Press (2006). p. 1483–579.

[110] Childs GV Unabia G Tibolt R Lloyd JM. Cytological factors that support nonparallel secretion of luteinizing hormone and follicle-stimulating hormone during the estrous cycle. Endocrinology (1987) 121:1801–13. 10.1210/endo-121-5-1801 2444429

[111] Lloyd JM Childs GV. Changes in the number of GnRH-receptive cells during the rat estrous cycle: biphasic effects of estradiol. Neuroendocrinology (1988) 48:138–46. 10.1159/000125001 2851748

[112] Childs GV Unabia G Lloyd J. Recruitment and maturation of small subsets of luteinizing hormone gonadotropes during the estrous cycle. Endocrinology (1992) 130:335–44. 10.1210/endo.130.1.1727707 1727707

[113] Childs GV Unabia G Lee BL Rougeau D. Heightened secretion by small and medium-sized luteinizing hormone (LH) gonadotropes late in the cycle suggests contributions to the LH surge or possible paracrine interactions. Endocrinology (1992) 130:345–52. 10.1210/endo.130.1.1727708 1727708

[114] Childs GV. Division of labor among gonadotropes. Vitam Horm (1995) 50:215–86. 10.1016/S0083-6729(08)60657-3 7709601

[115] Adams TE Nett TM Interaction of GnRH with anterior pituitary. III. Role of divalent cations, microtubules and microfilaments in the GnRH activated gonadotroph. Biol Reprod (1979) 21:1073–86. 10.1095/biolreprod21.5.1073 229920

[116] Schaeffer M Hodson DJ Lafont C Mollard P. Endocrine cells and blood vessels work in tandem to generate hormone pulses. J Mol Endocrinol (2011) 47:R59–66. 10.1530/JME-11-0035 21622530

[117] Childs GV. Growth hormone cells as co-gonadotropes: partners in the regulation of the reproductive system. Trends Endocrinol Metab (2000) 11:168–75. 10.1016/S1043-2760(00)00252-6 10856917

[118] Moriarty GC. Electron microscopic-immunocytochemical studies of rat pituitary gonadotrophs: a sex difference in morphology and cytochemistry of LH cells. Endocrinology (1975) 97:1215–25. 10.1210/endo-97-5-1215 1237396

[119] Moriarty GC Immunocytochemistry of pituitary glycoprotein hormones. J Histochem Cytochem (1976) 24:846–63. 10.1177/24.7.60435 60435

[120] Childs GV Unabia G Rougeau D. Cells that express luteinizing hormone (LH) and follicle-stimulating hormone (FSH) beta-subunit messenger ribonucleic acids during the estrous cycle: the major contributors contain LH beta, FSH beta, and/or growth hormone. Endocrinology (1994) 134:990–7. 10.1210/endo.134.2.8299592 8299592

[121] Clayton RN Solano AR Garcia-Vela A Dufau ML Catt KJ. Regulation of pituitary receptors for gonadotropin-releasing hormone during the rat estrous cycle. Endocrinology (1980) 107:699–706. 10.1210/endo-107-3-699 6249571

[122] Funabashi T Brooks PJ Weesner GD Pfaff DW Luteinizing hormone-releasing hormone receptor messenger ribonucleic acid expression in the rat pituitary during lactation and the estrous cycle. J Neuroendocrinol (1994) 6:261–6. 10.1111/j.1365-2826.1994.tb00581.x 7522739

[123] Savoy-Moore RT Schwartz NB Duncan JA Marshall JC. Pituitary gonadotropin-releasing hormone receptors during the rat estrous cycle. Science (1980) 209:942–4. 10.1126/science.6250218 6250218

[124] Syed M Cozart M Haney AC Akhter N Odle AK Allensworth-James M Ghrelin restoration of function in vitro in somatotropes from male mice lacking the Janus kinase (JAK)-binding site of the leptin receptor. Endocrinology (2013) 154:1565–76. 10.1210/en.2012-2254 23417423 PMC3602631

[125] Childs GV Akhter N Haney A Syed M Odle A Cozart M The somatotrope as a metabolic sensor: deletion of leptin receptors causes obesity. Endocrinology (2011) 152:69–81. 10.1210/en.2010-0498 21084451 PMC3033057

[126] Odle A Allensworth-James M Akhter N Syed M Haney A MacNicol M A sex-dependent tropic role for leptin in the somatotrope as a regulator of POU1F1 and POU1F1-dependent hormones. Endocrinology (2016) 157:3958–71. 10.1210/en.2016-1472 27571135 PMC5045503

[127] Odle AK Beneš H Melgar-Castillo A Akhter N Syed M Haney A Association of Gnrhr mRNA with the Stem Cell Determinant Musashi: A Mechanism for Leptin-Mediated Modulation of GnRHR Expression. Endocrinology (2017) 159:1–12. 10.1210/en.2017-00586 29228137 PMC5776477

[128] Kaiser UB Sabbagh E Katzenellenbogen RA Conn PM Chin WW. A mechanism for the differential regulation of gonadotropin subunit gene expression by gonadotropin-releasing hormone. Proc Natl Acad Sci U S A (1995) 92:12280–4. 10.1073/pnas.92.26.12280 8618885 PMC40340

[129] Kaiser UB Jakubowiak A Steinberger A Chin WW. Differential effects of gonadotropin-releasing hormone (GnRH) pulse frequency on gonadotropin subunit and GnRH receptor messenger ribonucleic acid levels in vitro. Endocrinology (1997) 138:1224–31. 10.1210/endo.138.3.4968 9048630

[130] Trajkovski M Hausser J Soutschek J Bhat B Akin A Zavolan M MicroRNAs 103 and 107 regulate insulin sensitivity. Nature (2011) 474:649–53. 10.1038/nature10112 21654750

[131] Guo L Chen C Shi M Wang F Chen X Diao D Stat3-coordinated Lin-28-let-7-HMGA2 and miR-200-ZEB1 circuits initiate and maintain oncostatin M-driven epithelial-mesenchymal transition. Oncogene (2013) 32:5272–82. 10.1038/onc.2012.573 23318420

[132] Agarwal V Bell GW Nam JW Bartel DP. Predicting effective microRNA target sites in mammalian mRNAs. Elife (2015) 4. 10.7554/eLife.05005 26267216 PMC4532895

[133] Fox RG Park FD Koechlein CS Kritzik M Reya T. Musashi signaling in stem cells and cancer. Annu Rev Cell Dev Biol (2015) 31:249–67. 10.1146/annurev-cellbio-100814-125446 26566113

[134] Szabat M Kalynyak TB Lim GE Chu KY Yang YH Asadi A Musashi expression in beta-cells coordinates insulin expression, apoptosis and proliferation in response to endoplasmic reticulum stress in diabetes. Cell Death Dis (2011) 2:e232 10.1038/cddis.2011.119 22113197 PMC3223700

[135] Ayrout M Simon V Bernard V Binart N Cohen-Tannoudji J Lombes M A novel non genomic glucocorticoid signaling mediated by a membrane palmitoylated glucocorticoid receptor cross talks with GnRH in gonadotrope cells. Sci Rep (2017) 7:1537. 10.1038/s41598-017-01777-2 28484221 PMC5431531

